# Complementary and alternative medicine usage among cardiac patients: a descriptive study

**DOI:** 10.1186/s12906-015-0610-y

**Published:** 2015-03-31

**Authors:** Mandreker Bahall

**Affiliations:** Arthur Lok Jack Graduate School of Business, Max Richards Drive, Champ Fleurs, Mount Hope, Trinidad, Trinidad and Tobago

**Keywords:** Cardiac patients, Complementary and alternative medicine, Side effects, Treatment

## Abstract

**Background:**

The use of complementary and alternative medicine (CAM) persists, despite the availability of conventional medicine (CM), modernisation, globalisation, technological advancement, and limited scientific evidence supporting CAM. People with cardiovascular diseases often use CAM, despite possible major adverse effects and lack of evidence supporting CAM claims. This study explored CAM use among cardiac patients, the types of CAM used, reasons and factors that influence its use, and the association between patient demographics and CAM use.

**Methods:**

This cross-sectional quantitative study was conducted using quota sampling to survey 329 public clinic adult cardiac patients within the South–West Regional Health Authority (SWRHA) of Trinidad and Tobago. From 1 July 2012 to 31August 2012, each participant completed questionnaires, after consenting to participate. Data analysis included χ^2^ tests and binary logistic regression.

**Results:**

One hundred eighty-five (56.2%; standard error [SE] = 2.74%) patients used CAM. Herbal medicine was the most common CAM (85.9%; SE = 2.56%), followed by spiritual therapy/mind-body systems (61.6%; SE = 3.58%), physical therapy/body manipulation (13.5%; SE = 2.51%), alternative systems (8.1%; SE = 2.01%), and other methods (3.8%; SE = 1. 41%). The patients believed that CAM promotes health and wellness (79.5%; SE = 2.97%), assists in fighting illness (78.9%; SE = 3.00%), addresses the limitations of CM (69.2%; SE = 3.56%), alleviates symptoms (21.6%; SE = 6.51%), costs less than CM (21.6 %, SE = 3.03), and has fewer adverse/damaging effects than CM (29.7, SE =3.36), or they were disappointed with CM (12.4%, SE = 2.42). Ethnicity and religion were associated with CAM usage, but only ethnicity was a useful predictor of CAM use.

**Conclusions:**

Complementary and alternative medicine use was high among cardiac patients (56.2%, SE = 2.74%), and associated with ethnicity and religion. Friends, family, and perceived mode of action influenced a patient’s use of CAM.

## Background

There is a high prevalence of complementary and alternative medicine (CAM) use, even with great advances in conventional medicine (CM). The global prevalence of CAM use is 9.8–76.0% [1]. The prevalence is estimated at 38.0% in the United States in adults 18 years and older [2], 51.8% in the United Kingdom [3], and 68.9% in Australia [4]. In Canada, 12.4% of people visit a CAM practitioner [5]. The prevalence of CAM use in Trinidad and Tobago is unknown, although it appears widespread. In 2001, the National Health Service (NHS) in London, England reported that British people invested £50 million in CAM [6]. Adult Americans pay $34 billion out-of-pocket costs annually [7]. For many decades, CAM practiced in Trinidad [8] represented a major (and sometimes the only) form of medical treatment because of the lack of available health care [9]. Nonconventional medicine use in Trinidad has its genesis many centuries ago in the form of traditional medicine, which represents nonconventional medical practices indigenous to the country of origin. Traditional medicine was practiced by Amerindians (e.g. Carib and Arawak) and later by migrants such as enslaved Africans, indentured East Indians, and Chinese immigrants who brought with them customs and practices that included “home medication/remedies” and unconventional medical practices [10]. The use of CAM remains prevalent in Trinidad, despite greater accessibility to free healthcare services, a better understanding of disease conditions, and widespread communication through electronic and print media. However, its usage is not necessarily the result of decreased effectiveness of CM [11]. Conventional medical treatment of cardiovascular diseases (CVDs) has improved patient outcomes in quality of life, decreased mortality, decreased morbidity, and increased life expectancy [12]. However, CVD mortality accounts for 33.7% of all deaths in the Americas, with Guyana and Trinidad and Tobago having the highest rates [13]. The prevalence of CAM use among cardiac patients with coronary artery disease is 4–63% [14]. However, no studies have been performed on CAM use among cardiac patients in Trinidad and Tobago. Clement et al. [15] found that 30.4% of asthmatic patients used herbal medicines.

### Public health relevance

The use of CAM continues, even though there is a lack of necessary evidence to support many of its claims [7,16]. However, a new paradigm shift has led to the incorporation of relevant and tested CAM and CM into “integrative medicine” [17].

Many people continue to gravitate to unregulated CAM practitioners who use therapies of questionable safety and efficacy, and may even be injurious to health [18]. Complementary and alternative medicine therapies can even replace conventional therapies. The increasing use and cost of CAM with favourable and unfavourable consequences have made it a major public health problem because it affects the lives of individuals and communities.

Public safety and ensuring that the claims of CAM can stand up to scientific scrutiny should be emphasised, according to a former Chief Medical Officer of Trinidad and Tobago [19]. The World Health Organization (WHO) Traditional Medicine Strategy of 2002–2005 emphasised four public health areas of CAM: (1) policy; (2) safety, efficacy, and quality; (3) access; and (4) rational use [20]. To encourage more rational, safe, and effective CAM usage, the expanding use of CAM—even among conventional physicians—must therefore be redefined with regard to its role in light of an expanding public health movement [21]. To achieve these goals, the Minister of Health of Trinidad and Tobago disclosed that laws were being drafted to regulate herbal practice [22]. However, to date, such laws have not been enacted.

This study will focus on the use of CAM among cardiac patients. More specifically, this study aimed to explore the types of CAM used, the reasons and influences for its use, benefits and outcomes and the association between patient demographics and CAM use.

## Methods

This cross-sectional study was conducted among cardiac clinic attendees within the South–West Regional Health Authority (SWRHA) of Trinidad and Tobago between 1 July 2012 and 31 August 2012. Quota sampling (i.e. successive sampling until the size of the sample is achieved) was used to select the participants. A total of 329 patients was the predetermined sample size required to estimate with a 5% margin of error the percentage of public cardiac clinic patients who use CAM [23]. To be eligible for participation, patients could not be confused (i.e. display problems with cognition or behaviour), as assessed by the student research assistant; had to be able to communicate verbally or in writing; and had to give consent. The data collection instrument was a self-completed questionnaire of 33 questions: seven questions on demographics; two questions on present cardiac condition; and the remaining questions on various aspects of CAM usage such as types, experiences, reasons, benefits, influences, effects and consequences, source, and access of CAM. Two medical students assisted participants who had difficulty understanding questions or who required clarification of questions.

Statistical analysis was conducted using SPSS, version 20 (Chicago, IL, USA) using descriptive methods and inferential methods. The descriptive methods included frequency distribution tables and graphs. Inferential methods included tests of equality of proportions, chi-squared tests of association between selected sociodemographic and other attribute variables and CAM use (e.g. Fisher’s exact test and McNemar’s test of paired proportions). Binary logistic regression was used to identify factors associated with CAM use among patients. Eight independent variables were used such as sex, marital status, ethnicity, educational level, employment status, religion, religiosity, and area of residence. All hypotheses were tested at the 5% level of significance. Ethical approval was obtained from the Clinical Governance and Ethics Committee of South–West Regional Health Authority on 25 May 2012.

## Results

**Figure 1:**
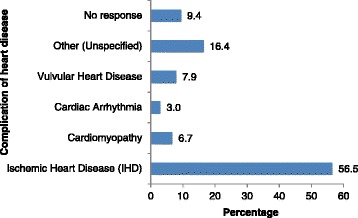
**Types of heart disease among the respondents.**

All 329 of the returned questionnaires were usable, and the reliability of the instrument (i.e. Cronbach α) was 0.896. Five participants were excluded because of difficulty communicating (i.e. verbalising or documenting their responses). Table 1 gives the frequency distribution of the patients’ sociodemographic variables. The respondents were predominantly male (n = 207; 62.9%); older than 60 years (n = 185, 56.2%); married (n = 264, 80.2%); of Indo-Trinidadian ethnic background (n = 238; 72.3%); had up to a primary school education (n = 213; 64.7%); and were of the Hindu religious faith (n = 180; 54.7%). In addition, most participants (n = 197; 59.9%) reported incomes ranging from US$385.00 to $769.00 per month. Ischaemic heart disease (IHD), which includes congestive cardiac failure, had the highest prevalence (n =186; 56.5%) among the respondents. Cardiac arrhythmia had the lowest prevalence (n = 10; 3.0%) (Figure 1). The heart disease for which 54 (16.4%) patients were treated was not stated (Figure 1).

**Table 1:** **Sociodemographic profile of the 329 patients**

**Variable**	**n**	**%**	**SE (** ***P*** **)**
Sex			
Male	207	62.9	2.67
Female	121	36.8	2.66
Age (y)			
<40	11	3.3	0.98
41–50	58	17.6	2.10
51–60	72	22.8	2.31
>60	158	56.2	2.74
Marital status			
Single	14	4.3	1.12
Married	264	8.2	1.51
Other	16	4.8	1.18
Unknown	12	3.6	1.03
Ethnicity			
Afro-Trinidadian	80	24.3	2.36
Indo-Trinidadian	238	72.3	2.47
Other (including mixed race)	11	3.3	0.98
Highest level of education			
Primary	213	64.7	2.63
Secondary	103	31.2	2.56
Other	2	0.6	0.43
Unknown	6	1.8	0.73
Employment status			
Employed	62	18.8	2.15
Unemployed	256	77.8	2.29
Unknown	11	3.3	0.98
Religion			
Hindu	180	54.7	2.74
Islam	27	8.2	1.51
Christian	75	22.8	2.31
Other	39	11.9	1.79
Unknown	8	2.4	0.84

SE = standard error.

Note: The denominators do not add up to 100% because of missing values and rounding off.

### Complementary and alternative medicine usage

Users of CAM accounted for 185 (56.2%) of the respondents. Among these, 115 (62.2%) respondents were male and 70 (37.8%) respondents were female. Most (98.9%) CAM users received CM. Drugs were the most common form of CM administered to the patients (93.0%). Complementary and alternative medicine and CM were used during the same period by 77.8% of CAM users. In addition, 78.9% of all respondents reported satisfaction with CM.

Table 2 compares the prevalence of selected types of heart disease between CAM users and CAM non-users. None of the differences between CAM users and non-users with respect to the prevalence of the various heart conditions was statistically significant. In addition, medicinal herbs was overall the most common type of CAM used (n = 159; 85.9%), followed by spiritual therapy/mind-body systems (n = 114; 61.6%), physical therapy/body manipulation (n = 25; 13.5%), alternative systems (n = 15; 8.1%), energy therapies (n = 3; 1.6%), and other (unspecified) forms of treatment (n = 7; 3.8%). The four leading CAM subtypes used were omega-3 fatty acids (54.6%), the B vitamins (50.4%), special diet/nutritional supplements (41.1%), and faith healing (54.1%). Some patients used more than one type of CAM simultaneously: 129 (69.7%) patients used two types of CAM and 44 (23.2%) patients used three types of CAM.

**Table 2:** **Chi-squared analysis results for heart disease distribution among CAM users and non-CAM users**

	**CAM Usage, n (%; SE [%])**		
**Type of heart disease**	**Yes (n = 185)**	**No (n = 144)**	***P*** **value for the chi-squared test**
Ischaemic heart disease	104 (62.7; 3.56)	82 (62.1; 4.04)	0.925
Cardiomyopathy	12 (7.2; 1.90)	10 (7.6; 2.01)	0.91
Cardiac arrhythmia	6 (3.6; 1.37)	4 (3.0; 1.42)	0.779
Valvular heart disease	13 (7.8; 1.97)	13 (9.8; 2.48)	0.544
Other	31 (18.7; 2.87)	23 (17.4; 3.16)	0.88

CAM = complementary and alternative medicine; SE = standard error.

The prevalence of medical herb use, which included unspecified herbs, was high for all reported heart disease types (Table 3), and ranged from 76.9% (valvular heart disease) to 100% (cardiac arrhythmia). This was followed by spiritual therapies with a prevalence ranging from 46.2% (valvular heart disease) to 100% (cardiac arrhythmia).

**Table 3:** **Type of CAM used, based on cardiac condition**

	**Type of CAM used, n (%; SE [%])**				
**Type of heart disease**	**Medicinal herbs**	**Spiritual therapies/mind–body systems**	**Physical therapies/body manipulations**	**Alternative systems**	**Other (including energy therapies)**
Ischaemic heart disease (n = 104)	94 (90.4; 2.89)	61 (58.7, 4.82)	15 (14.4; 3.44)	9 (8.7; 2.70)	0 (0.0)
Cardiomyopathy (n = 12)	11 (91.7; 7.96)	6 (50.0; 14.43)	1 (8.3; 7.96)	1 (8.3; 7.96)	0 (0.0)
Cardiac arrhythmia (n = 6)	6 (100.0; 0.00)	6 (100.0; 0.00)	3 (50.0; 20.41)	0 (0; 0.00)	0 (0.0)
Valvular heart disease (n = 13)	10 (76.9; 13.32)	6 (46.2; 15.77)	0 (0.0)	0 (0.0)	0 (0.0)
Other, not specified (n = 31)	24 (77.4; 7.51)	24 (77.4; 7.51)	0 (0.0)	0 (0.0)	6 (19.4, 7.10)

CAM = complementary and alternative medicine; SE = standard error.

### Associated factors and useful predictors of CAM use

**Figure 2:**
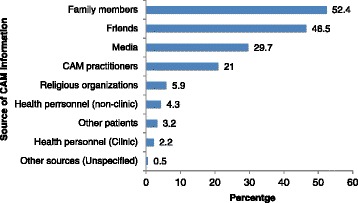
**Patients’ sources of information about complementary and alternative medicine. **CAM = complementary and alternative medicine.

The patients’ main sources of information about CAM were family members (52.4%; n = 97) and friends (46.5%; n = 86); whereas only 2.2% of patients (n = 4) received information from clinic personnel (Figure 2). Chi-squared tests of association between the socioeconomic variables and the use or non-use of CAM showed that CAM use was associated with ethnicity (χ^2^ = 18.20; df = 2; *P* ≤ 0.001) and a patient’s religion (χ^2^ = 12.02; df = 3; *P* = 0.007). Furthermore, binary logistic regression analysis identified ethnicity as the only useful predictor of whether a patient would use CAM (odds ratio [OR] = 1.962; *P* = 0.028; 95% confidence interval [CI], 1.094, 3.516) (Table 4).

**Table 4:** **Binary logistic regression model**

**Variable**	**OR**	***P*** **value**	**95% CI for OR**
Sex	1.104	0.712	(0.653, 1.868)
Marital status	0.550	0.079	(0.282, 1.073)
Ethnicity	1.962	0.024	(1.094, 3.516)
Education	0.999	0.943	(0.971, 1.027)
Employment	0.691	0.335	(0.326, 1.465)
Religion	0.848	0.325	(0.612, 1.177)
Religiosity	1.400	0.313	(0.729, 2.689)
Residence	1.289	0.372	(0.738, 2.251)

95% CI = 95% confidence interval; OR = odds ratio.

Reasons for CAM use were explored further by asking participants to list factors that either pushed them away from CM or enticed them towards CAM (Table 5). Among the leading “push” factors were the inadequacies of CM treatment (n = 128; 69.2%), whereas the leading “pull” factor was their belief that CAM promotes general health and wellness (n = 147; 79.5%).

**Table 5:** **The “push” and “pull” factors for using CAM in 185 patients**

**Push and pull factors for CAM usage**	**Number (n)**	**Percentage (%)**	**SE (%)**
Push Factors			
Inadequacies of CM	128	69.2	3.39
High cost of CM	40	21.6	3.03
Adverse effects and the toxic or damaging nature of CM	55	29.7	3.36
Disappointment with CM	23	12.4	2.42
Mechanistic nature of CM	6	3.2	1.29
Pull Factors			
CAM promotes general health and wellness	147	79.5	2.97
CAM specifically assists in fighting illness	146	78.9	3.00
CAM is helpful	137	74.1	3.22
Trying everything that can help	131	70.8	3.34
CAM improves physical well-being	117	63.2	3.55
CAM allows a user to relax and sleep	88	47.6	3.67
CAM directly treats complications	41	22.2	3.06
CAM improves psychological and emotional well-being	14	7.6	1.95
CAM gives users control of their treatment and faith	8	4.3	1.49
CAM is more in keeping with inner beliefs	7	3.8	1.41

CAM = complementary and alternative medicine; CM = conventional medicine; SE = standard error.

### Outcome and satisfaction with CAM

Most patients (n = 137; 74.1%) reported that CAM use resulted in positive outcomes and that they were generally satisfied with its usage. Less than two percent of users (n = 3; 1.6%) experienced a bad outcome and only a few users (n = 4; 2.2%) experienced complications. Good outcomes were most common (83.3%, n =12) among cardiomyopathy patients, followed by patients with IHD (82%, n = 104). Among the 155 CAM users, 8 (5.2%) patients were very satisfied; 133 (85.8%) patients were satisfied; and 14 (7.6%) patients were disappointed with the outcome of CAM. Most (84.9%) CAM users were satisfied with medical herbs, followed by spiritual therapies (74.5%). Twelve (7.7%) of 155 CAM users experienced an unwanted effect. Most (88.1%) CAM users reported they would encourage others to use alternative therapies.

## Discussion

The NCCAM defines complementary and alternative medicine as “a group of diverse medical and health care systems, practices, and products that are not generally considered part of conventional medicine” [24]. Complementary and alternative medicine includes herbs, dietary supplements, meditation, biofeedback, hypnosis, acupuncture, Ayurveda, homeopathy, naturopathy, Chinese medicine, chiropractic, massage, *tai chi*, yoga, electromagnetic therapy, kinesiology, *reiki*, and *qigong*. In the current study, which is the first conducted among cardiac patients in Trinidad, patients were not provided a definition of CAM. However, they were asked to choose from a wide list of options that captured nearly the entire spectrum of CAM.

The prevalence of CAM use among cardiac patients in this study was high (56.2%), males outnumbered females, and the number of Indo-Trinidadians was significantly higher than that of other ethnic groups. At least 69.7% of CAM users simultaneously used at least two types of CAM, and 23.2% of CAM users simultaneously used at least three types of CAM. In the United Kingdom in a group of West Midlands hospitals, the prevalence of CAM users among coronary artery disease (CAD) patients for the preceding 12 months was reportedly 31.7% [25], and in the state of Texas in the United States of America, the prevalence of CAM usage among cardiovascular patients is 54% [26]. Nicdao and Ai [27] found that the sociodemographic predictors of CAM use identified before cardiac surgery include income, religiosity, education, body mass index, employment, and congestive cardiac failure. Ethnicity and religion in this study were two factors associated with CAM use, but only ethnicity was predictive of CAM use.

The patients in this study recognised the perceived value of CAM, but most (77.8%) patients did not stop using CM. This may reflect a continued appreciation of the value of CM, fear of stopping CM, or lack of full trust in CAM. Complementary and alternative medicine usage is attributed to the increasing demand and expectations for more holistic and comprehensive care [28]. However, CAM practices depend on the social, cultural, economic, and traditional influences of societies [29]. As previously reported by similar studies, people were encouraged by one or more push or pull factors (Table 5). “Push” factors included dissatisfaction with conventional treatments, whereas “pull” factors included a desire for more holistic and “natural” approaches, and a greater philosophical congruence with CAM [30]. Complementary and alternative medicine is perceived as “natural” and “safe” [31], effective [32], and having fewer adverse effects [33]. Patients using CAM also experience a feeling of control, coping, and adjustment [34]. Bekke-Hassen et al. [35] found faith and motivational factors to be strong influences in CAM usage. As in this study, CAM was more likely to be used if patients held strong beliefs about traditional theories of health, illness, and remedies, or if family members encouraged them to use it [36].

Users of CAM have claimed better health and satisfaction. Its use has expanded because of the lack of regulations in Trinidad and Tobago [19], and because of support and encouragement, particularly from friends (46.5%), family (52.4%), CAM practitioners (21.1%), and mass media (29.7%). Such usage exists, even when there is little encouragement from health personnel (4.3%).

Complementary and alternative medicine is not as beneficial or safe as is proclaimed because its use may result in a significant cost financially or otherwise (e.g. by delaying treatment or by causing death). Its usage may have serious implications for patients and society by creating false hope, compounding adverse effects, and delaying CM treatment and utilisation of resources that could be channelled into evidenced-based practices. The cardiac conditions listed in Table 2 may require the use of blood thinners such as aspirin, heparin, warfarin, and clopidogrel, which may interact with many herbal products commonly used by cardiac patients. Herb–drug interactions can modify the actions of drugs (e.g., digoxin) that have a narrow therapeutic index or other commonly used drugs such as diuretics, beta-blockers, cholesterol-lowering drugs and amiodarone. Patients’ easy acceptance of natural products as “safe” has largely ignored the possibility of herb–herb interactions, herb–drug interactions, and herbal toxicity.

Herb–drug interactions include arrhythmias [37] or increased toxicity, particularly when drugs with a narrow therapeutic range such as digoxin or warfarin are coadministered with herbs [7]. Patients, CM practitioners, and CAM practitioners have to be mindful of the possibility of the increased risk of bleeding with ginkgo [38] and garlic (*Allium sativum* L) [39], both of which are used by cardiac patients, especially if these herbs are taken with blood thinning medications. However, claims of such adverse interactions have not been supported in other studies [40,41]. Folk remedies practiced in Trinidad can expose individuals to toxins that can be dangerous to health [42], and vigorous massage therapy can lead to negative adverse effects and should be avoided in patients with heart failure, kidney failure and bleeding disorders.

Rabito and Kaye [28] argue that CAM usage can lead to positive and significant benefits. This cannot be disregarded because in this study many of the most popular herbal medicines such as omega-3 fatty acids [43], CoQ10 [44], and fresh fruit and juices [45] have proven beneficial in reducing CAD. The B vitamins may be useful in preventing CAD; however, their value in CAD is unclear, according to some studies [46,47].

The high prevalence of CAM practiced concurrently with CM necessitates greater understanding, communication, and integration of both. In this study, most (71.9%, n = 133) patients were willing to disclose such information to their CM provider. The few patients who were unwilling to disclose such information believed their physician would stop their use (33%, n = 30); the remaining patients (60%, n = 30) believed that disclosure of information about CAM use was unnecessary or unimportant. This is in contrast to the findings of Liu et al. [48] who found that only 17% of patients disclosed CAM practices to their physician, but as many as 48% of patients were unprepared to discuss the topic at all. A study in the state of Texas in the United States revealed more than 50% of patients reported that they did not inform their cardiologists of CAM use [26]. Furthermore, more than 90% of physicians did not discuss CAM treatments with their patients [14]. There has thus been no meaningful integration of CAM and CM.

The limitations of this study include the selection of patients from a single public health cardiac clinic from South Trinidad. The attendees of the clinic tended to be of a lower socioeconomic status and educational level, which is not fully representative of the population. The sample was not randomised, which could increase bias. In addition, the questionnaire was not pilot-tested. Another weakness or limitation of the study is the chance of false-positive findings.

## Conclusions

The prevalence of CAM use was high among cardiac patients, despite evidenced-based guidelines for CVD management. This research highlights the widespread use of CAM and patients’ perception of its benefit. The study findings are that most users are driven to CAM for general health and well-being, for fighting illness, and for addressing the inadequacies of CM. Patients are prepared to use CAM and CM simultaneously. Patients occasionally stop CM and use CAM alone. Therefore, patients, the community, and CM and CAM practitioners need to have adequate information to guide patients in effective and appropriate usage, to minimise the risks of CAM usage, and to give greater empowerment to CAM users. This will also assist in providing safe and effective health care.

## Abbreviations

CAM: Complementary and alternative medicine
CM: Conventional medicine
CVD: Cardiovascular disease
IHD: Ischaemic heart disease
NCCAM: National Center for Complementary and Alternative Medicine
NHS: National Health Service
CAM: complementary and alternative medicine
